# The physical capability of community-based men and women from a British cohort: the European Prospective Investigation into Cancer (EPIC)-Norfolk study

**DOI:** 10.1186/1471-2318-13-93

**Published:** 2013-09-10

**Authors:** Victoria L Keevil, Shabina Hayat, Nichola Dalzell, Stephanie Moore, Amit Bhaniani, Robert Luben, Nicholas J Wareham, Kay-Tee Khaw

**Affiliations:** 1 Department of Public Health and Primary Care, University of Cambridge, Strangeways Research Laboratory, Wort’s Causeway, Cambridge CB1 8RN, UK; 2 MRC Epidemiology Unit, Institute of Metabolic Science, Addenbrooke’s Hospital, Cambridge, UK

**Keywords:** Ag(e)ing, Sarcopenia, Hand strength, Physical capability, Sex factors

## Abstract

**Background:**

The European Working Group for Sarcopenia in Older People (EWGSOP) published a case-finding algorithm for sarcopenia, recommending muscle mass measurement in older adults with low grip strength (women <20 kg; men <30 kg) or slow walking speed (≤0.8 m/s). However, the implications of adopting this algorithm into clinical practice are unclear. Therefore, we aimed to explore the physical capability of men and women from a British population-based cohort study.

**Methods:**

In the European Prospective Investigation into Cancer-Norfolk study, 8,623 community-based adults (48-92 years old) underwent assessment of grip strength, walking speed, timed chair stands and standing balance. The proportion of older men and women (≥65 years) fulfilling EWGSOP criteria for muscle mass measurement was estimated. Additionally, cross-sectional associations of physical capability with age and sex were explored using linear and logistic regression.

**Results:**

Approximately 1 in 4 older participants (28.8%) fulfilled criteria for muscle mass measurement with a greater proportion of women than men falling below threshold criteria (33.6% versus 23.6%). Even after adjustment for anthropometry, women were 12.4 kg (95% Confidence Interval [CI] 12.0, 12.7) weaker, took 12.0% (95% CI 10.0, 14.0) longer to perform five chair stands and were 1.82 (95% CI 1.48, 2.23) times more likely to be unable to hold a tandem stand for 10 seconds than men, although usual walking speed was similar. Physical capability was inversely associated with age and per year, walking speed decreased by 0.01 m/s (95% CI 0.01, 0.01) and grip strength decreased by 0.49 kg (men; 95% CI 0.46, 0.51) and 0.25 kg (women; 95% CI 0.23, 0.27). Despite this, there was still variation within age-groups and not all older people had low physical capability.

**Conclusions:**

Every effort to optimise functional health in later life should be made since poor function is not inevitable. However, if the EWGSOP sarcopenia case-finding algorithm is endorsed, large proportions of older people could qualify for muscle mass measurement which is not commonly available. Considering population ageing, further discussion is needed over the utility of muscle mass measurement in clinical practice.

## Background

Population ageing is a global phenomenon and of particular concern in the United Kingdom (UK) as those born in the post-war ‘baby boom’ approach retirement age [1]. Poor health in later life is not inevitable [2,3] and an important aspect of ‘healthy ageing’ is the maintenance of independent living. Physical capability encompasses the ability to perform essential physical tasks such as washing and dressing. Objective measures of physical capability such as grip strength, walking speed, timed chair stands and standing balance discern a range of function amongst non-disabled older adults [4] and lower physical capability has been associated with a higher risk of future institutionalisation, disability and death [5,6].

Physical capability measures have also been suggested as appropriate screening tools for sarcopenia. The European Working Group on Sarcopenia in Older People (EWGSOP) proposed that the presence of low muscle mass with either low muscle strength and/or low physical performance characterises the syndrome [7]. A case-finding algorithm for use in clinical practice proposed that older people (>65 years) with a walking speed of ≤0.8 m/s or with grip strength <20 kg (women) or <30 kg (men) should have an assessment of muscle mass. However, the service development and public health implications of this algorithm are unknown. A feasible and practical way to measure muscle mass accurately in this context has not been agreed. The EWGSOP recommend using Dual-Energy X-ray Absorptiometry (DXA), bioelectrical impedance analysis (BIA) or anthropometric methods (mid-upper arm or calf circumference and skinfold thickness). However, the accuracy of anthropometric methods is uncertain and neither DXA nor BIA are commonly available in routine clinical practice. The widespread use of DXA scanning has been limited by cost and although BIA is more affordable, as well as being feasible in a range of healthcare settings, use in clinical practice has been held back by concerns relating to the validity of measurements in patients e.g., those on diuretics. Additionally, BIA does not measure body composition directly but utilises the different conductance of adipose versus muscle tissue to estimate fat and fat-free mass using prediction equations also incorporating anthropometric measures (e.g., height and weight or BMI). Currently, there is insufficient evidence that prediction equations using both anthropometry and BIA are superior in terms of body composition prediction to equations using height and weight (or BMI) alone [8]. Thus, there has been little impetus to change practice and re-train healthcare staff to incorporate BIA measurement into routine patient assessments.

Furthermore, few population-based studies have published strength and performance measures by age-group and gender, making it difficult to estimate how many older people are likely to fall below thresholds necessitating muscle mass measurement. This is apparent from several recent descriptive meta-analyses of physical capability [9-11]. Data available for synthesis was often from small, convenience samples and of the population-based studies included, most were from the USA. It is difficult to extrapolate findings from these studies to other countries since absolute levels of physical capability vary between populations [12].

Several UK epidemiological studies have measured physical capability. However, many are birth cohorts [13-16], are small in size [13,17] or are single sex [18]. This means that some are only able to report across limited age ranges (at least cross-sectionally), have small numbers within each age and sex strata or can only report on one sex. In the European Investigation into Cancer (EPIC)-Norfolk study, a prospective cohort study, the most recent phase obtained measures of functional performance. At the third health examination (EPIC-Norfolk 3 or 3HC) physical capability was measured objectively in 8,623 community-based men and women aged 48-92 years. This data collection phase enables EPIC-Norfolk to become the largest, single cohort study to report sex-specific values across a range of age-groups for grip strength, walking speed, timed chair stands and standing balance in a population-based sample of British people.

This paper aims to use this information to estimate the impact on healthcare services of applying the EWGSOP recommendations for sarcopenia case-finding, in terms of muscle mass assessment. Cross-sectional associations of physical capability with age and sex will also be explored.

## Methods

### Study design and population

EPIC-Norfolk is a prospective cohort study established as part of an international collaboration [19]. Between 1993 and 1997, 30,445 community-based men and women aged 40-74 years old and registered with participating general practices in Norfolk were enrolled (response rate 43%). The cohort at baseline was similar to participants from the Health Survey for England [20].

The latest phase of the study, EPIC-Norfolk 3 (2004-2011), focused on aspects of health pertinent to older age and 8,623 participants returned for the 3HC. Signed, informed consent was obtained at baseline and renewed at the 3HC. The study was approved by the Norfolk Local Research Ethics Committee and the East Norfolk and Waveney NHS Research Governance Committee.

In order to evaluate the effects of attrition since baseline, participants who attended both the first and third health examinations were compared to those who attended the first but not the third. Those who did not return were more likely to have been smokers and older, shorter, and heavier with higher blood pressure at baseline [21].

### Physical capability measures

All measures were taken in the EPIC-Norfolk 3 clinic by trained research nurses following standardised protocols. Physical performance measures were based on those used in the Established Populations for the Epidemiologic Study of the Elderly (EPESE) [6].

Hand grip strength was measured as a marker of general muscle strength [22] using a hand-held dynamometer (Smedley’s Dynamometer, Scandidact, Kvistgaard, Denmark). Participants performed the test standing and two measures with each hand were recorded, alternating between hands [12]. Participants who were unable to stand performed the test sitting. The maximum strength recorded was used in analyses.

Usual walking speed was measured along a 4 metre (m) course. Participants wore comfortable shoes or walked in bare feet, could use a walking aid and began the test from a standing start. Timing began when the participant’s foot first crossed the start line after the command ‘Go’ and stopped when the finish line was crossed, although participants kept walking towards a red line 1 m further. Two trials of the test were performed and walking speed was estimated by dividing 4 m by the average of the two times recorded.

A straight-backed chair, placed against a wall, with a hard seat and standard height was used for all chair stands. Participants were asked to sit with their feet on the floor and both arms folded across their chests. Timing began as soon as the command ‘Stand’ was given and stopped when the participant straightened their body after the fifth rise. During the test the examiner counted each rise out aloud. Encouragement to perform the test quickly was given before but not during the test. Participants only performed the test once and for analysis the natural log of the time recorded was used.

The ability to stand for 10 seconds unassisted with feet in a side-by-side, semi-tandem and tandem position was assessed. These standing positions are progressively more challenging and those unable to complete the easier positions did not continue. For analysis, results were dichotomised into those able or unable to hold a tandem stand for 10 seconds.

### Covariates

Height was measured to the nearest 0.1 cm using a stadiometer (Chasmores, UK). Weight was measured to the nearest 0.1 kg using digital scales (Tanita UK Ltd, Middlesex, UK).

### Statistical analyses

Prior to analysis the dataset was cleaned to remove implausible or incongruous results (see Additional file 1). Physical capability measures are presented by 5 year age-group and sex using means (standard deviation, SD), medians (inter-quartile range, IQR) and proportions (frequency, %). The proportion of participants ≥65 years old fulfilling EWGSOP criteria necessitating muscle mass measurement was calculated.

Associations of age and sex with physical capability were evaluated using linear regression with the exception of standing balance, for which logistic regression was used. The resulting regression coefficients represent the mean difference in grip strength (kg), the mean difference in walking speed (m/s), the percentage difference in time to complete five chair stands (×100, %) and the odds of being unable to hold a tandem stand for 10 seconds, per unit change in the independent variable. Height and weight were also included in regression models to adjust results for anthropometric factors. Since height and weight are correlated, sex specific standardised residuals for weight were calculated after regression of weight on height. Hereafter, ‘weight’ refers to weight adjusted for height [23].

An interaction term between age and sex was also entered into regression models to evaluate whether sex modifies the association of physical capability with age. Additionally, the distributions of strong versus weak and fast versus slow (in terms of usual walking speed) men and women were evaluated across 10 year age groups. Cut points for low grip strength and slow walking speed were <30 kg (men)/ < 20 kg (women) and ≤0.8 m/s, as per the EWGSOP criteria.

Differences in age and sex between participants with and without physical capability data were assessed using T-tests and chi-squared tests.

## Results

**Figure 1:**
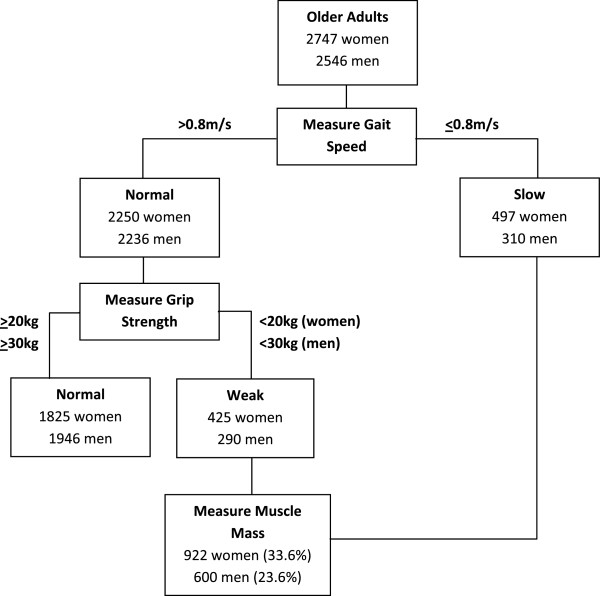
**Part of the EWGSOP algorithm for sarcopenia case-finding **[7]**.** The EWGSOP algorithm for sarcopenia case-finding has been adapted to show the number of older participants (aged ≥65 years old) in EPIC-Norfolk 3 who fell below physical capability thresholds necessitating muscle mass measurement.

8,623 men and women aged 48-92 years attended the 3HC (Table 1). Of these, 5,426 participants were ≥65 years old and 5,293 (97.5%) had sufficient data to apply the EWGSOP sarcopenia case-finding algorithm. 922 women (33.6%) and 600 men (23.6%) fulfilled criteria necessitating muscle mass measurement (Figure 1). In total, this equated to just over 1 in 4 participants (28.8%, n=1,522).

**Table 1:** The characteristics of men and women participating in EPIC-Norfolk 3

**Characteristic**	**Men (N=3861)**	**Women (N=4762)**
**Mean (sd)**		
Age (years)	69.4 (8.1)	68.1 (8.0)
Height (cm)	173.5 (6.7)	160.5 (6.2)
Weight (kg)	81.7 (12.3)	68.6 (12.9)
**Frequency, % (n)**		
Smoking status		
Never smoker	38.3 (1,469)	59.3 (2,800)
Social class		
I-IIINM	64.5 (2,472)	67.2 (3,165)
Physical activity		
Inactive	37.4 (1,422)	37.2 (1,748)
Moderately inactive	25.1 (954)	32.2 (1,513)
Moderately active	18.8 (713)	16.9 (796)
Active	18.8 (714)	13.6 (641)
Educational level		
No qualification	22.2 (857)	29.7 (1,412)
O level	9.8 (378)	13.6 (648)
A level	48.0 (1,851)	41.2 (1,959)
Degree or equivalent	20.1 (774)	15.6 (742)

*sd* standard deviation, *cm* centimetres, *kg* kilograms, *N* number, *I-IIINM* I-III Non-Manual.

Physical capability by age group and sex is shown in Table 2. The associations of each physical capability measure with age and sex are described in more detail below.

**Table 2:** Maximum grip strength, chair stands, usual walking speed and standing balance by sex and 5-year age group in men and women of EPIC-Norfolk 3

	**Physical capability measure**					
	**Men**			**Women**		
**Age (Years)**	**N**	**Mean (sd)**	**Range**	**N**	**Mean (sd)**	**Range**
**Maximum grip strength, kg**						
48–54	123	47.7 (7.7)	33-72	185	28.6 (5.5)	8-40
55–59	262	45.0 (8.4)	10-67	468	27.0 (4.8)	7-43
60–64	867	42.7 (7.2)	17-68	1241	26.0 (5.3)	6-42
65–69	802	40.4 (7.2)	9-65	967	24.8 (5.1)	5-44
70–74	752	37.8 (7.0)	13-57	810	23.4 (5.0)	3-40
75–79	583	35.0 (6.3)	15-53	592	21.5 (4.6)	8-36
80–84	328	32.0 (6.7)	10-49	304	19.8 (4.8)	8-36
85–92	95	27.8 (6.2)	12-48	94	17.3 (4.3)	5-26
**Usual walking speed, m/s**						
48–54	129	1.23 (0.2)	0.65-1.96	185	1.24 (0.2)	0.46-2.15
55–59	265	1.23 (0.2)	0.34-2.03	473	1.22 (0.2)	0.40-1.91
60–64	868	1.22 (0.2)	0.46-1.96	1240	1.16 (0.2)	0.33-1.83
65–69	801	1.16 (0.2)	0.14-2.16	975	1.11 (0.2)	0.23-1.85
70–74	755	1.10 (0.2)	0.28-1.96	822	1.03 (0.2)	0.26-3.14
75–79	588	1.02 (0.2)	0.13-2.12	604	0.95 (0.2)	0.08-1.78
80–84	331	0.93 (0.2)	0.26-1.61	300	0.88 (0.3)	0.13-1.53
85–92	91	0.83 (0.2)	0.34-1.48	94	0.78 (0.2)	0.29-1.30
**Time to complete 5 chair rises, s**						
		**Median (IQR)**			**Median (IQR)**	
48–54	123	9.7 (8.2, 12.1)	4.1-26.0	174	10.3 (8.4, 12.1)	5.6-34.0
55–59	260	10.3 (8.2, 12.4)	4.3-22.6	451	10.3 (8.7, 12.7)	4.5-28.0
60–64	828	10.6 (9.0, 12.9)	4.1-35.1	1148	11.2 (9.5, 13.2)	5.0-31.4
65–69	744	11.1 (9.4, 13.6)	5.1-27.8	883	11.8 (10.0, 14.2)	4.2-34.7
70–74	700	12.1 (10.1, 14.8)	5.2-59.5	721	13.0 (10.9, 15.8)	5.8-57.4
75–79	488	13.4 (11.4, 16.1)	5.7-33.1	496	13.8 (11.4, 16.8)	5.3-37.6
80–84	232	13.7 (11.5, 16.9)	4.9-32.3	215	14.6 (12.0, 18.4)	7.3-56.4
85–92	52	15.1 (13.4, 19.0)	9.6-25.6	57	16.3 (13.6, 20.9)	7.5-35.3
**Standing balance, % (n) able to hold tandem stand for 10 s**						
		**Frequency, % (n)**			**Frequency, % (n)**	
48–54	129	96.9 (125)		185	99.5 (184)	
55–59	268	98.9 (265)		480	95.0 (456)	
60–64	874	96.2 (841)		1257	92.8 (1,166)	
65–69	805	93.7 (754)		986	90.7 (894)	
70–74	762	90.9 (693)		829	81.8 (678)	
75–79	593	83.1 (493)		615	72.4 (445)	
80–84	334	70.7 (236)		310	56.5 (175)	
85–92	96	43.8 (42)		100	50.0 (50)	

*N* number, *sd* standard deviation, *kg* kilograms, *s* seconds, *m/s* metres/ second, *%* frequency.

### Age

An inverse association between age and physical capability was observed. After adjustment for height and weight, per year of older age, men were 0.49 kg (95% CI 0.46, 0.51) weaker, walked 0.012 m/s (95% CI 0.011, 0.013) more slowly, took 1.6% (95% CI 1.4, 1.7) longer to perform five chair stands and were 1.14 (95% CI 1.12, 1.16) times more likely to be unable to hold a tandem stand for 10 seconds (Table 3). The same trends were observed in women who were 0.25 kg (95% CI 0.23, 0.27) weaker, walked 0.013 m/s (95% CI 0.012, 0.014) more slowly, took 1.6% (95% CI 1.5, 1.8) longer to perform five chair stands and were 1.13 (95% CI 1.11, 1.14) times more likely to be unable to hold a tandem stand for 10 seconds.

**Table 3:** Association of age with physical capability

**Physical capability measure**	**N**	**Regression coefficient**^**a **^**(95% Confidence interval)**	
		**Unadjusted**	**Adjusted for height &weight**
**Maximum grip strength, kg**			
**Men**	3804	−0.54* (−0.57, −0.51)	−0.49* (−0.51, −0.46)
**Women**	4653	−0.30* (−0.32, −0.28)	−0.25* (−0.27, −0.23)
**Usual walking speed, m/s**			
**Men**	3822	−0.012* (−0.013, −0.011)	−0.012* (−0.013, −0.011)
**Women**	4698	−0.014* (−0.015, −0.013)	−0.013* (−0.014, −0.012)
**Timed chair stands, ln(s)**			
**Men**	3425	0.014* (0.013, 0.016)	0.016* (0.014, 0.017)
**Women**	4145	0.015* (0.014, 0.016)	0.016* (0.015, 0.018)
**Standing balance, OR**			
**Men**	3850	1.14* (1.12, 1.16)	1.14* (1.12, 1.16)
**Women**	4753	1.12* (1.11, 1.13)	1.13* (1.11, 1.14)

^a^Regression models evaluated the mean difference in maximum grip strength and usual walking speed and the relative difference in chair stand time per year of advancing age. For standing balance the regression coefficient represents the odds of being unable to hold a tandem stand for 10 seconds per year of advancing age.

*P value <0.001.

*N* number, *kg* kilograms, *m/s* metres/ second, *Ln(s)* natural logarithm(seconds), *OR* odds ratio.

**Figure 2:**
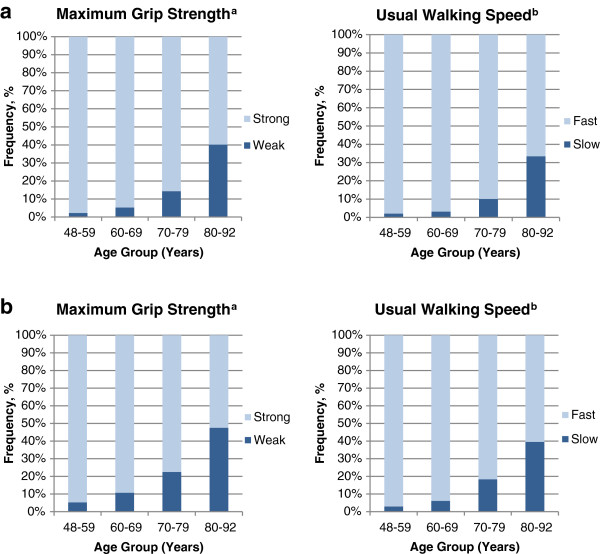
**The range of physical capability within 10-year age groups in men (2a) and women (2b). **^**a**^Weak participants had grip strengths of <30 kg (men) or <20 kg (women). ^**b**^Slow participants had a usual walking speed of ≤0.8 m/s.

Accordingly, Figure 2 shows that the prevalence of slow walking speed and low grip strength increases with age. However, amongst older participants there is still a range of function, with over half in the oldest age group not meeting criteria for slow walking speed or low grip strength and performing better than some younger participants.

### Sex

In unadjusted analyses and after adjustment for age men were more physically capable than women. In analyses adjusted for age, height and weight, women were 12.4 kg (95% CI 12.0, 12.7) weaker, took 12.0% (95% CI 10.0, 14.0) longer to perform five chair stands and were 1.82 (95% CI 1.48, 2.23) times more likely to be unable to hold a tandem stand for 10 seconds than men. However, no difference in usual walking speed was observed between sexes (Table 4).

**Table 4:** The physical capability of women compared to men and evidence for an interaction between age and sex

		**Regression coefficient (95% Confidence interval)**			
**Physical capability measure**	**N**	**Women compared to men**^**a**^		Interaction term: Age* Sex^**b**^	
		**Adjusted for age**	**Adjusted for age, height &weight**	**Adjusted for age**	**Adjusted for age, height &weight**
**Maximum grip strength, kg**	8457	−15.32* (-15.58, -15.06)	−12.35* (-12.72, -11.98)	0.24* (0.21,0.27)	0.24* (0.21,0.27)
**Usual walking speed, m/s**	8512	−0.05* (-0.06, -0.04)	−0.01 (-0.02, 0.01)	−0.001** (-0.002,-0.0002)	−0.001** (-0.002,-0.0001)
**Timed chair stands, ln(s)**	7570	0.05* (0.03, 0.06)	0.12* (0.10, 0.14)	−0.001 (-0.001,0.002)	0.001 (-0.001,0.002)
**Standing balance, OR**	8603	1.85* (1.61, 2.12)	1.82* (1.48, 2.23)	0.98 (0.97, 1.00)	0.98 (0.97, 1.00)

^a^Regression models evaluated the mean difference in maximum grip strength and usual walking speed and the relative (percentage) difference in chair stand time in women compared to men. In terms of standing balance, the odds of being unable to hold a tandem stand for 10 seconds in women compared to men is represented.

^b^Regression models evaluated age-sex interactions with age, sex, height and weight included in the model as well as the interaction term. Positive regression coefficients represent a diminishing gap between women and men as age increases whereas negative coefficients indicate a widening gap, if men are more physically capable than women in the youngest age-group.

*P value <0.001; **P value <0.05.

*N* number, *kg* kilograms, *m/s* metres/ second, *Ln(s)* natural logarithm(seconds), *OR* odds ratio.

### Does sex modify associations with age?

An interaction between age and sex was observed for maximum grip strength such that the absolute gap between men and women tapers as age increases (β= 0.24 kg; 95% CI 0.21, 0.27) (Table 4). This did not change after adjustment for height and weight. However, there was no evidence that gender modified the associations of standing balance or timed chair stands with age and only a weak interaction term was observed for walking speed (β= -0.001 m/s; 95% CI -0.002, -0.0001). With respect to usual walking speed, the regression coefficient for the interaction term was very small and indicated that the decline in walking speed across age-groups was slightly steeper in women than men (see also Additional file 2).

**Figure 3:**
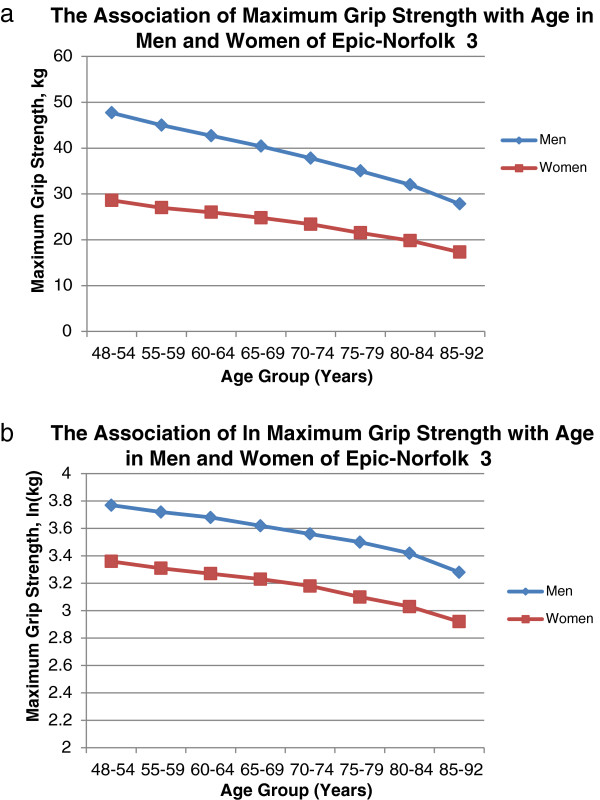
**Absolute mean maximum grip strength (kg) (3a) and relative mean maximum grip strength (natural log of grip strength, lnkg) (3b) in men and women by 5-year age group.** Linear regression models estimate the mean maximum grip strength **(3a)** and the natural log of the mean maximum grip strength **(3b)** per age category using the least squares method. Models were fitted with an interaction term between age-group and sex (both categorical variables) and age-group, sex, height and weight were also included as individual covariates. Figure 3a shows that the slope of the cross-sectional association of grip strength with age is much steeper in men than women. Accordingly, when age-group is entered as a continuous variable into the model, an interaction between age-group and sex is observed (β=1.20 kg, p<0.001). Figure 3b represents the relative grip strength by age-group. The cross-sectional association between age and grip strength now appears similar in both sexes. When age-group is entered as a continuous variable into the model, the interaction term between age-group and sex indicates that the gap between men and women decreases by 0.7% per 5 years (β=0.007, p=0.01), a much smaller effect size.

To further explore associations with grip strength, a linear regression model was used to generate the least square mean grip strength, adjusted for height and weight, by 5 year age-groups in men and women. Both maximum grip strength and the natural logarithm of maximum grip strength were entered as dependent variables in separate models, in order to evaluate the cross-sectional associations of absolute and relative grip strength with age respectively. Absolute grip strength plotted against age-group revealed a much steeper decline with age for men compared to women, as expected (Figure 3a). However, when the natural logarithm of maximum grip strength was used, this difference attenuated substantially although not completely (Figure 3b).

### Missing physical capability measurements

150 (1.7%), 102 (1.2%) and 1,051 (12.2%) participants did not have grip strength, walking speed or timed chair stand measurements respectively. Compared to those with grip strength measurements, those without were older (70.4 yrs vs 68.7 yrs, p=0.01) and more likely to be female (67.3% vs 55.0%, p=0.003). Similar trends were seen with missing data for walking speed and timed chair stands (data not shown).

## Discussion

To our knowledge, this is the largest cohort study to report age and sex-specific values for objective measures of physical capability in British men and women. Using this data, we found that just over 1 in 4 community-based, older adults in our cohort would meet criteria for muscle mass measurement, if the EWGSOP algorithm for sarcopenia case-finding was applied. This could pose significant challenges to healthcare services, since measurement of muscle mass is not widely available in routine clinical practice.

Few studies have reported the numbers of older people falling below strength and performance thresholds suggested in the EWGSOP algorithm. Data from a recent report of another UK cohort revealed 22% of community-dwelling participants (aged 59-73 years old) enrolled in a physical performance sub-study met criteria for muscle mass measurement [24]. This is comparable to our findings, with the slightly higher proportion reported here explained by the older ages of the participants included, with many older than 73 years. We found that age was strongly and inversely associated with physical capability, consistent with other reports [25-31]. Unadjusted results showed that usual walking speed declined by 0.01 m/s and grip strength by 0.54 kg (men) and 0.30 kg (women) per year. Thus, as age increases more people are likely to fall below the suggested physical capability thresholds necessitating muscle mass assessment and it is not surprising that our older cohort had higher numbers of participants in this category. The inverse association between physical capability and age and the ageing nature of many populations also suggests that the number of people likely to meet criteria necessitating muscle mass measurement is only likely to rise over the coming decades.

We found that a greater proportion of women than men fell below physical capability thresholds, of significance given the increasing numbers of older women in the population. This was despite the use of sex-specific grip strength criteria and reflects the greater functional dependency experienced by older women. Consistent with this, women had weaker grip strength than men, took longer to perform 5 chair stands and had poorer standing balance even after adjustment for age and anthropometry. This concurs with existing literature [25-31] and adds to the observed male-female health-survival paradox [32], since women are known to have longer life expectancies than men but lower physical capability is a factor associated with a higher risk of death. One possible explanation is that men decline faster than women and this is important for future health. Cross-sectional associations of absolute grip strength with age did show a steeper decline in men than women, but this difference substantially attenuated when the relative change with age was assessed. This suggests that the steeper decline of absolute strength in men is partly explained by the higher strength of young men compared to young women. However, a small age-sex interaction was still observed when relative change in strength was examined and mixed results have been reported from longitudinal studies examining this interaction [33-35]. Therefore, further work in this area is needed.

The age and sex associations reported here are remarkable in their similarity to those published from the HALCyon study group, after harmonisation and meta-analysis of independent participant data from eight, smaller and heterogenous UK cohorts (see Additional file 3) [28]. Meta-analysis of individual participant data is increasingly advocated [36]. However, large or well-known studies are more likely to be invited to share data creating possible bias and it is still uncertain how much studies can differ and yet still be combined in pooled analyses. Despite this, it is interesting that the size and direction of associations are very similar in this single, large British cohort to those reported in the meta-analysis.

The EWGSOP consensus definition [7] has provided a much needed opportunity to standardise sarcopenia research in order to progress efforts to untangle its underlying pathogenesis and develop potential therapies. Sarcopenia identified using this definition has been associated with poor self-reported health and physical function [24] and with future increased risk of mortality [37]. However, the EWGSOP definition has been little scrutinised. A surprisingly low prevalence of sarcopenia (0.9%) was reported when using this definition in Finnish older women [38] and on examination of the algorithm’s component parts the authors found no association between functional performance and low muscle mass. The predictive power of low muscle mass has been debated previously. Associations with future disability and mortality [39] have been less consistent and strong [26,40,41] than associations between gait speed or muscle strength and future health-related outcomes [4,5,42,43].

Maintenance of physical function with increasing age is important and although we found strong inverse associations between age and physical capability, we also report considerable variation in physical capability within older age-groups. Therefore, it is important to understand the factors leading to this heterogeneity and to develop interventions to mitigate the disability associated with older age and its considerable healthcare cost [44]. However, currently suggested interventions for sarcopenia, such as resistance exercise training, are effective in a wide range of older people including those who do not have confirmed low muscle mass [45]. Considering this and the less strong and consistent associations of muscle mass with future health outcomes, compared to tests of strength and function, whether muscle mass measurement is necessary in the wider clinical setting warrants debate.

The EWGSOP suggested criteria for low muscle strength and slow walking speed also deserve appraisal. These criteria are based on a report from a population-based study in Italy (n=1,020; age 20-85+ years) [26]. However, these cut-off points may not be suitable for all populations, considering differences in absolute physical capability levels reported between populations of different countries [46,47] even when part of the same study [12].

It is also possible that levels of physical capability reported here are unusually high or unusually low, limiting our ability to assess the potential impact of applying the EWGSOP algorithm. Other UK and European population-based studies reporting physical capability levels are summarised in Additional file 4: Tables S1 and S2. On average participants in our study walked more quickly than participants from other UK cohorts but were similar in terms of grip strength, particularly to participants from the English Longitudinal Study of Ageing (ELSA). With respect to the faster usual walking speed in our cohort, it could be that selective attrition of participants with poorer health, during the 20 years of follow-up, has resulted in a sample less representative of the general population than the cohort at baseline [21]. However, absolute physical capability levels are difficult to compare between studies, due to differences in measurement protocols [48,49]. Overcoming this barrier, we measured grip strength using the same dynamometer and protocol to ELSA, whose participants were originally enrolled in the Health Survey for England. Therefore, when using comparable measurement protocols, the physical capability of our cohort is similar to a nationally representative sample.

The EPIC-Norfolk study and measurements taken at the 3HC have some limitations. Associations between age and physical capability are explored using cross-sectional data. Thus, we cannot be certain that we are examining age-related changes in physical capability. Additionally, strength and performance measures were not reported in all participants and data was more likely to be missing in older, female participants. This bias could explain the tapering of the gender gap in maximum grip strength with advancing age. Against this is the relatively low volume of missing data for grip strength (1.7%) and the considerable attenuation of the age-sex interaction when the relative change in grip strength was calculated.

Nevertheless, EPIC-Norfolk 3 comprises a large number of participants from the general population at baseline, with a wide age-range across both sexes. Participants have been examined under standardised conditions by trained research nurses using validated measurement techniques to examine a comprehensive range of physical capability measures. Additionally, any truncation of the cohort due to selective attrition of the frailest members is likely to make some of our findings an underestimation.

## Conclusions

Every effort should be made to optimise physical capability in later life since poor function is not inevitable. However, this report of the range of physical capability in community-based men and women of a British cohort reveals the significant potential impact of sarcopenia case-finding on healthcare services, in terms of muscle mass assessment. Considering that populations are ageing, this is only likely to increase and further work is needed to translate sarcopenia research into practical clinical policies that will benefit patients. EPIC-Norfolk 3 will be a useful and rich resource for future investigation of conditions pertinent to ageing.

## Competing interests

The authors declared that they have no competing interests.

## Authors’ contributions

Study concepts and design: SH, ND, RL, KTK, NW; Participant recruitment and data collection: SM, AB, SH, ND, RL, KTK, NW; Analysis and interpretation of data: VK, KTK. Manuscript: VK drafted and wrote the manuscript with review and contributions from co-authors. All authors have read and approved the manuscript.

## Pre-publication history

The pre-publication history for this paper can be accessed here:

http://www.biomedcentral.com/1471-2318/13/93/prepub

## Supplementary Material

Additional file 1 Data cleaning: description of the data cleaning methods. Click here for file

Additional file 2 The association of usual walking speed and timed chair stands performance with age in men and women of EPIC-Norfolk 3: graphical illustration of the associations of usual walking speed and timed chair stands performance with age group and sex. Click here for file

Additional file 3 The physical capability of women compared to men and evidence for an interaction between age and sex: results presented by the HALCyon study group using harmonised data from several British cohorts. Click here for file

Additional file 4: Tables S1 and S2 European population-based studies reporting usual walking speed and grip strength by age group and sex: tabulated data from relevant population based studies identified in a literature review. Click here for file
