# Supplementary material for: The physical capability of community-based men and women from a British cohort: the European Prospective Investigation into Cancer (EPIC)-Norfolk study
Source: BMC Geriatr. 2013 Sep 10;13:93. doi: 10.1186/1471-2318-13-93 (PMC3846689; doi:10.1186/1471-2318-13-93)
Supplement: Additional file 1 — Data cleaning: description of the data cleaning methods. [file 1471-2318-13-93-S1.docx]

**The physical capability of community-based men and women from a British cohort: The European Prospective Investigation into Cancer (EPIC)-Norfolk Study**

**Additional File 1**

**Data Cleaning**

Grip strength values which differed by >20kg between trials in the same hand (6 participants) and all values more than 100kg (4 values) were excluded from analysis. Prior to calculation of walking speed, times to walk 4 metres of less than 1 second and more than 60 seconds (4 values) or where the two trials of the timed walk differed by more than two times each others’ value (12 participants) were excluded. If only one trial was recorded, walking speed was calculated using this time. With respect to the timed chair stands, values of less than 4 seconds and more than 60 seconds (31 values) were excluded. Participants who were unable to stand for 10 seconds in the side by side or semi-tandem positions were coded as ‘unable to hold a tandem stand for 10 seconds’, even if they had progressed on to perform tandem stands in deviation from the study protocol.
