# Supplementary material for: The physical capability of community-based men and women from a British cohort: the European Prospective Investigation into Cancer (EPIC)-Norfolk study
Source: BMC Geriatr. 2013 Sep 10;13:93. doi: 10.1186/1471-2318-13-93 (PMC3846689; doi:10.1186/1471-2318-13-93)
Supplement: Additional file 2 — The association of usual walking speed and timed chair stands performance with age in men and women of EPIC-Norfolk 3: graphical illustration of the associations of usual walking speed and timed chair stands performance with age group and sex. [file 1471-2318-13-93-S2.docx]

**The physical capability of community-based men and women from a British cohort: The European Prospective Investigation into Cancer (EPIC)-Norfolk Study**

**Additional File 2**

The least square mean usual walking speed or timed chair stands performance, after adjustment for height and weight, by 5 year age group and sex.

*the actual time taken in seconds was used to generate this graph rather than the natural logarithm of the time. This makes the associations with age and sex easier to understand in this illustrative format.
