# Supplementary material for: The physical capability of community-based men and women from a British cohort: the European Prospective Investigation into Cancer (EPIC)-Norfolk study
Source: BMC Geriatr. 2013 Sep 10;13:93. doi: 10.1186/1471-2318-13-93 (PMC3846689; doi:10.1186/1471-2318-13-93)
Supplement: Additional file 3 — The physical capability of women compared to men and evidence for an interaction between age and sex: results presented by the HALCyon study group using harmonised data from several British cohorts. [file 1471-2318-13-93-S3.docx]

**The physical capability of community-based men and women from a British cohort: The European Prospective Investigation into Cancer (EPIC)-Norfolk Study**

**Additional File 3**

The physical capability of women compared to men and evidence for an interaction between age and sex: results presented by the HALCyon study group using harmonised data from several British cohorts [1]

| **Physical Capability Measure** | **Regression Coefficient Adjusted for Body Size^a^**  **(95% Confidence Interval)** | | | |
| --- | --- | --- | --- | --- |
|  | **N** | **Women Compared to Men** | **N** | **Interaction term: Age*Sex** |
| **Maximum Grip Strength, kg** | 14,213 | -12.62 (-13.90, -11.34) | 10,840 | 0.25 (0.22,0.28) |
| **Usual walking Speed, m/s** | 8,246 | -0.02 (-0.06, 0.01) | 7,705 | -0.002 (-0.004,-0.0001) |
| **Timed Chair Stands ln(s)** | 10,754 | 0.11 (0.05, 0.17) | 8,035 | -0.01 (-0.02,0.01) |
| **Standing Balance** | 12,838 | 1.48 (1.27, 1.72) | 9,980 | 1.02 (1.00, 1.03) |

^a^height and weight where possible or body mass index

Reference

1. Cooper R, Hardy R, Aihie Sayer A, Ben-Shlomo Y, Birnie K, Cooper C, Craig L, Deary IJ, Demakakos P, Gallacher J, McNeill G, Martin RM, Starr JM, Steptoe A, Kuh D: **Age and gender differences in physical capability levels from mid-life onwards: the harmonisation and meta-analysis of data from eight UK cohort studies.** *PLoS One* 2011, **6**:e27899.
