# Supplementary material for: The physical capability of community-based men and women from a British cohort: the European Prospective Investigation into Cancer (EPIC)-Norfolk study
Source: BMC Geriatr. 2013 Sep 10;13:93. doi: 10.1186/1471-2318-13-93 (PMC3846689; doi:10.1186/1471-2318-13-93)
Supplement: Additional file 4: Tables S1 and S2 — European population-based studies reporting usual walking speed and grip strength by age group and sex: tabulated data from relevant population based studies identified in a literature review. [file 1471-2318-13-93-S4.docx]

**The physical capability of community-based men and women from a British cohort: The European Prospective Investigation into Cancer (EPIC)-Norfolk Study**

**Additional File 4**

**Table S1** European population-based studies reporting walking speed by age group and sex

| **Study**  **(Reference)** | **Location** | **N** | **Distance** | **Walking**  **aid** | **Age Groups**  **(Years)** | **Mean (sd) Walking speed (m/s)** | |
| --- | --- | --- | --- | --- | --- | --- | --- |
|  |  |  |  |  |  | **Men** | **Women** |
| HAS  [1] | UK | 289 | 3m | Yes | 70-74  75-79  80-84 | 0.93 (0.2)  0.84 (0.2)  0.79 (0.2) | 0.80 (0.2)  0.79 (0.2)  0.43 (0.1) |
| HCS  [1] | UK | 2,300 | 3m | Yes | 60-64  65-69  70-74 | 0.97 (0.1)  0.94 (0.2)  0.91 (0.2) | 0.95 (0.2)  0.91 (0.2)  0.87 (0.2) |
| ELSA  [1] | UK | 5,499 | 2.4m | Yes | 60-64  65-69  70-74  75-79  80-84  85-89  90+ | 1.00 (0.3)  0.95 (0.3)  0.88 (0.3)  0.81 (0.2)  0.72 (0.3)  0.63 (0.2)  0.56 (0.3) | 0.95 (0.3)  0.91 (0.3)  0.82 (0.3)  0.74 (0.2)  0.62 (0.2)  0.55 (0.2)  0.47 (0.2) |
| HSE 2005  [2] | UK | 3,145 | 2.4m | Yes | 65-69  70-74  75-79  80-84  85+ | 1.0 (0.3)  0.9 (0.3)  0.9 (0.3)  0.8 (0.3)  0.7 (0.2) | 0.9 (0.3)  0.9 (0.3)  0.8 (0.3)  0.7 (0.2)  0.5 (0.2) |
| SardiNIA  [3] | Sardinia, Italy | 3,872 | 4m | unknown | <35  35-54  55-74  75+ | 1.2 (0.2)  1.2 (0.2)  1.1 (0.2)  0.9 (0.2) | 1.2 (0.2)  1.1 (0.2)  0.9 (0.2)  0.7 (0.2) |
| InChianti  [4] | Italy | 1,228 | 4m | Yes | <65  65-74  75-84  85+ | 1.36 (0.2)  1.21 (0.2)  1.06 (0.2)  0.81 (0.3) | 1.30 (0.2)  1.09 (0.2)  0.89 (0.2)  0.71 (0.3) |
| Umea 85+ Study  [5] | Sweden | 238 | 2.4m | Yes | 85  90  95+ | 0.47 (0.2-0.7)*  0.51 (0.3-1.0)  0.54 (0.2-0.8) | 0.52 (0.3-0.8)*  0.41 (0.2-0.7)  0.41 (0.2-0.6) |

*95% Confidence Intervals around the mean

HAS: Hertfordshire Ageing Study; HCS: Hertfordshire Cohort Study; ELSA: English Longitudinal Study of Ageing; HSE: Health Survey for England; InChianti: Invecchiare in Chianti; N: number, m: metre; sd: standard deviation; m/s: metres per second

**Table S2**  European population-based studies reporting grip strength by age group and sex

| **Study**  **(Ref)** | **Location** | **N** | **Dynamometer** | **Protocol** | **Age Group**  **(Years)** | **Mean (sd) Grip Strength (kg)** | |
| --- | --- | --- | --- | --- | --- | --- | --- |
|  |  |  |  |  |  | **Men** | **Women** |
| HAS  [1] | UK | 716 | Jamar | Max grip strength after 3 attempts in each hand | 60-64  65-69  70-74 | 40.7 (5.9)  37.8 (7.6)  37.0 (5.8) | 24.1 (5.8)  22.7 (5.1)  20.9 (5.5) |
| HCS  [1] | UK | 2,987 | Jamar | Max grip strength after 3 attempts in each hand | 55-59  60-64  65-69  70-74 | 49.3 (4.7)  45.3 (7.2)  43.3 (7.6)  41.9 (7.2) | -  27.3 (5.8)  26.4 (5.7)  25.0 (5.5) |
| ELSA  [1] | UK | 7,553 | Smedley | Max grip strength after 3 attempts in each hand | 50-54  55-59  60-64  65-69  70-74  75-79  80-84  85-89  90+ | 48.2 (7.9)  45.7 (8.5)  42.9 (9.1)  40.2 (8.3)  37.9 (7.4)  34.0 (7.5)  30.2 (7.4)  28.1 (7.2)  26.2 (5.7) | 28.4 (5.8)  27.2 (5.9)  25.8 (6.1)  24.5 (5.4)  22.6 (5.6)  20.6 (5.3)  17.9 (5.2)  17.2 (4.5)  14.6 (4.9) |
| InChianti  [6] | Italy | 1030 | Hydraulic hand-held dynamometer | The best of 2 attempts in each hand were averaged | 20-29  30-39  40-49  50-64  65-74  75-84  85+ | 61.1 (57-65)*  56.4 (52-61)  53.2 (49-58)  49.1 (45-53)  39.2 (38-41)  31.8 (30-34)  27.1 (23-31) | 35.6 (32-39)*  34.3 (32-36)  31.8 (30-34)  27.1 (25-29)  22.2 (21-23)  19.3 (18-21)  14.5 (13-16) |
| Health2006  [7] | Denmark | 3471 | Jamar | Max grip strength of dominant hand after 3 attempts | 19-29  30-39  40-49  50-59  60-72 | 50.5 (8.2)  52.7 (7.7)  52.9 (7.1)  48.9 (7.0)  44.4 (6.8) | 32.4 (5.1)  34.4 (5.8)  33.7 (5.4)  30.2 (5.5)  26.7 (4.8) |

*95% Confidence Intervals around the mean

HAS: Hertfordshire Ageing Study; HCS: Hertfordshire Cohort Study; ELSA: English Longitudinal Study of Ageing; InChianti: Invecchiare in Chianti; N: number, sd: standard deviation; kg: kilogram

**References**

1. Cooper R, Hardy R, Aihie Sayer A, Ben-Shlomo Y, Birnie K, Cooper C, Craig L, Deary IJ, Demakakos P, Gallacher J, McNeill G, Martin RM, Starr JM, Steptoe A, Kuh D: **Age and gender differences in physical capability levels from mid-life onwards: the harmonisation and meta-analysis of data from eight UK cohort studies.** *PLoS One* 2011, **6**:e27899.

2. Asher L, Aresu M, Falaschetti E, Mindell J: **Most older pedestrians are unable to cross the road in time: a cross-sectional study.** *Age Ageing* 2012, **41**:690–4.

3. Tolea MI, Costa PT, Terracciano A, Griswold M, Simonsick EM, Najjar SS, Scuteri A, Deiana B, Orrù M, Masala M, Uda M, Schlessinger D, Ferrucci L: **Sex-Specific Correlates of Walking Speed in a Wide Age-ranged Population**. *Journal of Gerontology: Psychological Sciences* 2010, **65B**:174–184.

4. Shumway-cook A, Guralnik JM, Phillips CL, Coppin AK, Ciol MA, Bandinelli S, Ferrucci L: **Age-associated Declines in complex Walking Task Performance: the Walking InChianti Toolkit**. *J Am Geriatr Soc* 2007, **55**:58–65.

5. Von Heideken Wagert P, Gustafson Y, Lundin-Olsson L: **Large variations in walking, standing up from a chair, and balance in women and men over 85 years: an observational study.** *Aust J Physiother* 2009, **55**:39–45.

6. Lauretani F, Russo CR, Bandinelli S, Bartali B, Cavazzini C, Di Iorio A, Corsi AM, Rantanen T, Guralnik JM, Ferrucci L: **Age-associated changes in skeletal muscles and their effect on mobility: an operational diagnosis of sarcopenia.** *J Appl Physiol* 2003, **95**:1851–60.

7. Aadahl M, Beyer N, Linneberg A, Thuesen BH, Jørgensen T: **Grip strength and lower limb extension power in 19-72-year-old Danish men and women: the Health2006 study.** *BMJ open* 2011, **1**:e000192.
